# Dopamine system: manager of neural pathways

**DOI:** 10.3389/fnhum.2013.00854

**Published:** 2013-12-09

**Authors:** Simon Hong

**Affiliations:** McGovern Institute for Brain Research and Department of Brain and Cognitive Sciences, Massachusetts Institute of TechnologyCambridge, MA, USA

**Keywords:** habenula, motivation, learning, prediction error, vigor, L-dopa, energy, Parkinson’s disease

## Abstract

There are a growing number of roles that midbrain dopamine (DA) neurons assume, such as, reward, aversion, alerting and vigor. Here I propose a theory that may be able to explain why the suggested functions of DA came about. It has been suggested that largely parallel cortico-basal ganglia-thalamo-cortico loops exist to control different aspects of behavior. I propose that (1) the midbrain DA system is organized in a similar manner, with different groups of DA neurons corresponding to these parallel neural pathways (NPs). The DA system can be viewed as the “manager” of these parallel NPs in that it recruits and activates only the task-relevant NPs when they are needed. It is likely that the functions of those NPs that have been consistently activated by the corresponding DA groups are facilitated. I also propose that (2) there are two levels of DA roles: the *How* and *What* roles. The *How* role is encoded in tonic and phasic DA neuron firing patterns and gives a directive to its target NP: how vigorously its function needs to be carried out. The tonic DA firing is to provide the needed level of DA in the target NPs to support their expected behavioral and mental functions; it is only when a sudden unexpected boost or suppression of activity is required by the relevant target NP that DA neurons in the corresponding NP act in a phasic manner. The* What* role is the implementational aspect of the role of DA in the target NP, such as binding to D1 receptors to boost working memory. This *What* aspect of DA explains why DA seems to assume different functions depending on the region of the brain in which it is involved. In terms of the role of the lateral habenula (LHb), the LHb is expected to suppress maladaptive behaviors and mental processes by controlling the DA system. The demand-based smart management by the DA system may have given animals an edge in evolution with adaptive behaviors and a better survival rate in resource-scarce situations.

## Introduction

Since the seminal work of Carlsson et al. (1958), the scientific community has gathered an enormous amount of data about dopamine (DA) and its actions in the brain and on behavior. The predominant theme that has emerged is the DA neurons’ ability to represent reward/punishment and motivation. There are numerous excellent and detailed reviews on the roles of DA (e.g., Schultz, 2007; Bromberg-Martin et al., 2010a; Salamone and Correa, 2012) so I will limit this article to my own opinion.

## Good management in technology and in biology

It is evident that technology has learned from biology, making machines through reverse engineering. One of the things that technology has adopted from biology is adaptive behavior. For example, recent CPUs of computers have evolved to become more adaptive and energy efficient. Instead of running at full speed all the time, as was the case in the past, now most of their modules go idle when there is no demand of them from the user. When the user demands a function, only the responsible module wakes up to meet the demand (Hübner and Becker, 2010; Panda et al., 2010). Also, when user demands can be handled in a leisurely manner, the CPU’s clock speed remains low; when demands become high, the CPU cranks up the speed to meet the demand. This was an inevitable evolution due to the explosive demand of mobile gadgets, such as smart phones and tablets, which are powered by batteries. As the gadgets became more powerful, so did the demand for power. The limitations of battery capacity have forced engineers to make the machines more energy efficient. Animals face the same challenge of energy efficiency. For example, an elk wintering with a limited food source often needs to dig the snow-covered frozen ground for grazing. In this situation, the elk needs to find and consume enough food to compensate for the loss of energy used for digging and digesting, not to mention withstanding the harsh cold weather; at the same time, the elk should limit unnecessary energy consuming activities to avoid death (Gates and Hudson, 1981; Christianson and Creel, 2009). I hypothesize that the brain implements a very efficient resource management system by recruiting and activating NPs only when they are needed, and also by tuning the activity level of mental and behavioral modules depending on the demand, similarly to the CPUs explained above. I suggest that the midbrain DA system is suited for this management role of deciding which NPs should be active, and to what degree, at a given moment. In terms of learning, it is likely that the elk has learned, in his lifetime and in evolution, where to find food and how to dig through snow via trials and errors. It is likely that the DA evaluation circuit around the basal ganglia (Hong et al., 2011) helps fine-tune the behavior of the animal to survive.

## Dopamine system: manager of NPS with “How” and “What” roles

**Figure 1:**
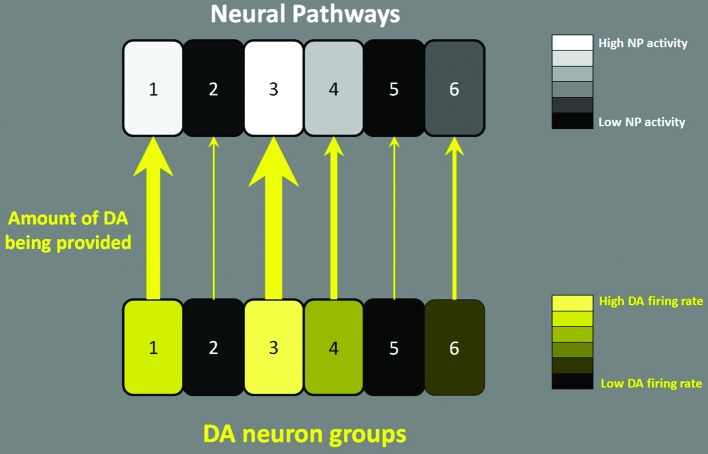
***How* role of DA neuronal firing.** Six separate NPs (e.g., 1: working memory NP, 2: some motor NP etc.) are illustrated. In the face of an event that requires an action, DA neurons fire to activate appropriate target NPs. According to the mentioned hypothesis, the firing rate of DA neurons (either in tonic firing or phasic busting) translates into the concentration of DA in the target and controls the level of activity of the target NPs. The level of lightness of the squares in the bottom row indicates the firing rate of DA neurons in different functional groups. The level of lightness of the squares in the top row indicates the activity level of the NPs. The thickness of the arrows indicates the amount of DA being provided to the target NPs; the thicker the arrows, the more DA provided. In the example above, NPs 1 and 3 are very active and are supported by the high level of DA coming from the corresponding DA neuron groups in the midbrain.

It has been suggested that largely parallel cortico-basal ganglia-thalamo-cortico loops exist to control different aspects of behavior (Alexander et al., 1986). I hypothesize that midbrain DA neurons are organized in a way to activate these parallel NPs to make them switch from their idling or maintenance state to a working state. Consistent with this hypothesis, Kolmac and Mitrofanis (1998) reported that the branches of DA neurons appear to target functionally homogeneous restricted thalamic and striatal subdivisions, therefore subpopulations of DA neurons potentially modulating specific NPs. According to this hypothesis, some groups of DA neurons are more active to make their target circuits work while other groups of DA neurons do not respond either because their target NPs are not needed at that moment or because the DA level in the target region is already sufficient to meet the activity level of these NPs. This description can be restated as follows: The midbrain DA system can be viewed as the manager of NPs, with a *How* (directive)** role, recruiting and activating the task-relevant NPs only when they are needed. The directive** (of how energetically carry out the NP function) of the DA system is encoded in the firing patterns (tonic, phasic) of DA neurons. Figure 1 illustrates this concept, in which the firing activities of DA neuron groups translate into the concentration of DA in the target (Venton et al., 2003) and control the levels of activity of the target NPs. Consistent with this hypothesis, a recent study (Howe et al., 2013) shows that even when the animal is motivated, the level of DA in the striatum is non-homogeneous; some parts increase and other parts decrease. In addition to this *How* role, DA has a *What* role: at the target NP, DA is utilized to support the NP’s intrinsic function, such as a behavioral action or any necessary mental process. I suggest that this is the reason why DA seems to assume different roles (*What* roles) depending on the region of the brain where it is involved. This *What* role hypothesis does not specify how DA needs to be utilized by the target neural circuit; the detailed usage of DA is left in the hands of the target local circuit for its own function. These two roles (*How* and *What*) can be exemplified as follows. Suppose there is a NP controlling working memory. Given a situation, for example, when a person’s date verbally lets him know his/her phone number, at this moment, a sudden surge of DA hits this NP and helps to hold the information (vigorous execution; *How* role). In this situation, the role of DA in the target NP is to promote working memory by blocking noise from disrupting the acquired information by mobilizing D1 receptors (*What* role; Sawaguchi and Goldman-Rakic, 1991). Given another situation, for example, when someone hears a phone number from a TV commercial, at this moment there is no DA surge for the NP, and he/she barely remembers the number for a second (non-vigorous execution; *How* role). In the above two situations, the role of DA in the target NP (binding to D1 receptors to boost working memory; *What* role) remains the same, but the way the function is carried out is quite different because DA is provided in different temporal patterns instructing the working memory function to be carried out in different ways (*How* role).

## Changing “How” directives: phasic firing supplements tonic firing when unexpected need arises

**Figure 2:**
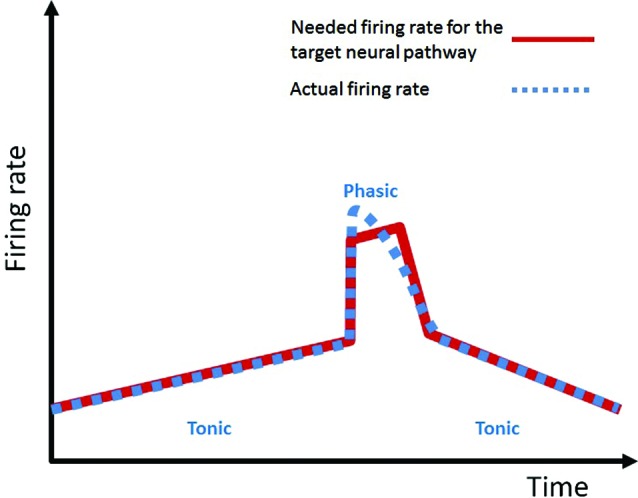
**“Demand-Supply” concept of DA firing activity.** The tonic (slow temporal scale: good at slow variation of firing over time, bad at instantaneous jump or dip) and phasic (fast temporal scale: good at instantaneous jump or dip, bad at slow variation over time) components of DA firing modes are utilized to meet the requested DA firing rate pattern (in multiple time scales) for the target neural pathway.

A big hurdle for the theory of DA going forward beyond reward and motivation has been the seemingly less-than-impressive modulation of activity of DA neurons by mundane activities including movements (Schultz, 1997). It has been described that most phasic DA neuron activations/suppressions happen when an unexpected event occurs (Schultz, 1997). I hypothesize that (a) the phasic activation or suppression of DA activity occurs because an event has occurred unexpectedly, requiring an unplanned, sudden, and often exaggerated action; and that (b) a large portion of DA roles in the target NPs can be accomplished by the tonic level of DA. Consistent with this hypothesis, tonic modulation of DA neuron firing during movements has been well documented (see Figure 4 in Schultz et al., 1983; Romo and Schultz, 1990). As the authors in these articles emphasize, tonic DA activity usually preceded movements and did not show any direct relationship with movement. These results are consistent with the current hypothesis of DA because a tonic DA level can be preemptively provided before the anticipated behavior. Tonic firing of DA neurons has been shown to be responsible for the baseline tonic level of DA concentration within the striatum (e.g., 10–20 nM within the striatal region (Keefe et al., 1993)). I hypothesize that this tonic DA level is to maintain a certain level of DA in the target NPs (Keefe et al., 1993) to support expected behavioral and mental functions. It is only when a sudden unplanned boost or suppression of activity in the target NP is required, DA neurons act in a phasic manner. This account is in accordance with the findings by Beierholm et al. (2013) in which the tonic DA level, manipulated by L-DOPA, correlated with the vigor of the subject’s response. Thus the tonic level of DA in the NPs can provide the vigor needed for planned routine activities; it is only when a sudden boost or suppression of vigor is needed that the phasic DA action is mobilized to quickly change the activity level of the NPs. A summary diagram of this DA demand in a target NP and the supply by the DA neuronal activity is shown in Figure 2. The tonic DA firing mode exhibits slow variation across time and is unable to exhibit instantaneous jumps or dips; therefore it operates on a slow temporal scale. The phasic mode is bad at slow variation of spiking across time and good at a sudden jumps or dips; therefore it operates on a fast temporal scale. To accomplish the needed temporal profile of DA firing pattern (e.g., the red line in Figure 2 has multiple scales: a slow temporal variation, and also a rapid variation), the DA neuron utilizes multiple scales (slow scale: tonic, fast scale: phasic) of firing modes.

There have been several accounts describing the tonic mode of DA neuron activation (e.g., Romo and Schultz, 1990; Goto et al., 2007; Bromberg-Martin et al., 2010b; Howe et al., 2013). However the tonic mode has not received as much attention as the phasic one. One of the reasons for the infrequent accounts in the literature may be that it is difficult to notice the subtle changes of spike counts in DA neurons. The discovery of the gradual modulation of DA neurons by Bromberg-Martin et al. (2010b) is a good example in which no one in the lab had realized there was a slow variation of DA firing rate across time, until a careful re-analysis of past data. Bromberg-Martin et al. (2010b) reports that when the monkey in the experiment feels that the next trial is overdue, neurons in the lateral habenula (LHb) and DA neurons increased and decreased, respectively, their tonic firing rates proportional to the delay. These gradual increments and decrements of firing rate may reflect a gradual form of motivation, i.e., a *How* directive to the target NPs. I predict that if the monkey somehow suddenly realized that the next trial was quite delayed, instead of slowly feeling the delay as was the case in the actual trials, the LHb and DA activity would be phasic, providing a different directive.

## Manager listens to multiple scale feedbacks

**Figure 3:**
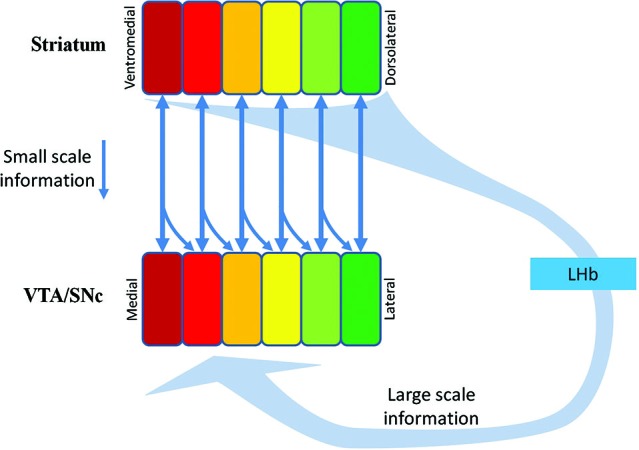
**Multiple-scale feedback inputs from the NPs in the striatum.** DA neurons in the VTA/SNc receive small-scale feedback information from their target NPs (the vertical downward arrows), and large-scale feedback information via the striatum→GPb→LHb→RMTg→DA projection. The scope of the pan-in-pan-out of the large scale information is unknown. The squares in Striatum indicate parallel NPs. The squares in VTA/SNc indicate functional groups of DA neurons supplying DA inputs to the counterpart NPs. SNc, Substantia nigra compcata; GPb, globus pallidus border neurons; LHb, lateral habenula; RMTg, rostromedial tegmental nucleus.

While the midbrain DA system may be the manager, its actions are controlled by numerous feedback paths coming from its target areas, particularly from the striatum. As the spiral organization (Haber et al., 2000) suggests, each little portion of the DA area along the medio-lateral swath of midbrain is bi-directionally connected with a specific part of the ventral or dorsal striatum, thus giving rise to numerous parallel loops, with adjacent loops often overlapping. While we do not know how the striatum-dopamine parallel loops work, it is likely that this striatum to DA feedback is quite local (Kolmac and Mitrofanis, 1998; Haber et al., 2000). This may be a mechanism for the DA neuron to gather information from a specialized NP. In addition to this small-scale feedback from a local target striatal NP, there seems to be a larger scale feedback as well. According to the work by Hong and Hikosaka (2008) and Hong et al. (2011), and the paper appearing in this issue) the LHb receives massive projections from the ventral and dorsal striatum via globus pallidus border (GPb) neurons, and influences DA neurons via the GABAergic neurons in the rostromedial tegmental nucleus (RMTg; Barrot et al., 2012). This indirect pathway, which funnels the inputs from the striatum to the midbrain DA area, seems to reach many DA neurons along the medio-lateral axis of the midbrain (Hong et al., 2011). The scope of this pan-in-pan-out is so far unknown (Hong et al., 2011, included only the reward-responding neurons in the DA area as DA neurons for analysis). Figure 3 illustrates the flow of feedback to the DA neurons. It is interesting that while the DA projections to the striatum target both the striosome and matrix (Prensa et al., 2009), most of the feedback from the striatum seems to originate from the striosome part of the striatum (Watabe-Uchida et al., 2012; Hong and Hikosaka, 2013, the accompanying paper in this issue). In summary, DA neurons receive “local expert’s” feedback from their striatal target NPs, and also receive a large-scale feedback from the LHb, possibly representing the “consensus” of the NPs. The scopes of these small scale (direct projection from the striatum) versus large-scale (indirect inputs from the LHb) inputs for each DA neuron need further study. It is interesting that while the nature of the small scale-input is unknown, the large-scale feedback seems to represent reward prediction error or instantaneous level of motivation (Hong et al., 2011).

As is presented here, the current theory of DA does not assume any homunculus manager in the midbrain that has a dictatorial power. Instead, the manager is composed of more or less independent groups of DA neurons, each group having its own source of local feedback and also a common feedback shared with many other groups. It is important to note that the contribution of DA neuron firings to the concentration of DA in a brain region (Venton et al., 2003) is just a part of the whole story. For example, a recent study shows that some GABAergic neurons in the ventral tegmental area (VTA) project to the nucleus accumbens (NAc) and suppress the cholinergic interneurons in the NAc, possibly enhancing the effect of DA in learning (Brown et al., 2012). Also, there are inputs from the thalamus to the striatum that could increase the efflux of DA from dopaminergic axonal terminals (Parsons et al., 2007; Threlfell et al., 2012), and the local configuration of the neural circuit also affects the efflux of DA (Rice et al., 2011). These mechanisms bypass the spiking action of midbrain DA neurons. These facts indicate that some managerial roles of the midbrain DA system may be shared with other brain regions.

## Motivation influences the manager’s “How” directive

Current hypotheses assume that there are many DA neuron groups serving their target NPs, each group possibly acting more or less independently. The fact that there are many DA neurons in the midbrain seemingly encoding reward and motivation may appear contradictory to this hypothesis. According to literature (e.g., Matsumoto and Hikosaka, 2009a) there are many DA neurons in the medial part of the substantia nigra pars compacta (SNc) and VTA, encoding the level of motivation of the subject. As the recording site moves more toward the lateral part of the SNc, fewer and fewer DA neurons respond to motivation-related events (Matsumoto and Hikosaka, 2009b). Nonetheless, it is true that there are many DA neurons seemingly encoding motivation in the SNc and VTA (Hong and Hikosaka, 2011b). The fact that the ventral striatum, including NAc, which is known for its role in goal directed behavior (Mogenson et al., 1980; Sesack and Grace, 2010), gets its DA inputs from the medial part of the SNc and VTA (Haber et al., 2000; Ikemoto, 2007) may partially explain why there are more motivation-related DA neurons in this area. Also the impression of prevalent activation of DA neurons by motivation (e.g., Fiorillo et al., 2013) may be due to the inherent relationship between motivation and action; increased motivation tends to make the subject mobilize many more parts of the body and thoughts to achieve the motivating goal. A detailed description of this relationship can be found in previous reviews (e.g., Salamone and Correa, 2012). In one example, Matsumoto and Hikosaka (2009a) have revealed that while many DA neurons found on the lateral side of the SNc responded to reward-related events these neurons also responded to punishing events, therefore prompting the researchers to classify those neurons as salience coding neurons rather than motivation coding. This shows that while many DA neurons across the SNc are seemingly encoding motivation, a careful examination may reveal differentiated roles among the DA neurons. Considering the conclusion by Hong et al. (2011) that the LHb modulates many DA neurons across the midbrain, it is possible that the dual encoding of salience and motivation by individual DA neurons along the lateral part of the SNc is a result of the dual (small and large scales) feedback explained above. More specifically, the salience part of DA firing may be a result of a NP-specific input while the motivation related signal is due to the broader influence of the LHb.

Considering the fact that the striosomal projection onto the DA neurons (small-scale feedback) is GABAergic, it is possible that the NP-specific excitatory inputs come from a different source. Some of the candidates of excitatory inputs are the PPTg (Kobayashi and Okada, 2007; Hong and Hikosaka, 2011b) and dorsolateral tegmental area (Sesack and Grace, 2010; Lammel et al., 2012). The PPTg receives inputs from the output pathways of the dorsal striatum, i.e., the globus pallidus internal segment (Shink et al., 1997) and from the cortex (Matsumura et al., 2000) therefore potentially receiving the information of individual NP activities. Also it is known that PPTg is one of the prominent excitatory input sources to midbrain DA neurons (Charara et al., 1996; Geisler et al., 2007). Considering these two facts, it is possible that the PPTg delivers excitatory inputs to target DA neurons in a NP-specific manner. Non-NP-specific accounts of striosome-mediated inhibition and PPTg-mediated excitation of DA neurons have been proposed (Brown et al., 1999; Suri and Schultz, 1999; Kobayashi and Okada, 2007).

## Different types of dopamine neurons

If we assume that DA neurons exist to activate appropriate NPs when they are needed, then it is natural to expect to find different groups of DA neurons, some increasing their firing rate and others not responding or even being suppressed during a given situation ( Figure 1). A few studies exist on this issue: Matsumoto and Hikosaka (2009a) describe one group of neurons responding to an air puff by increasing its firing rate and another group by decreasing its firing rate. The Ungless group (Brischoux et al., 2009) describes two groups of DA neurons, one group phasically increasing and the other decreasing its firing rate in response to noxious footshocks. Chiodo et al. (1980) also showed similar results. Steinfels et al. (1983) examined the activity of DA neurons in freely moving cats. Interestingly, many DA neurons were most active when the cat was actively walking. Also, some DA neurons were responsive to visual and auditory stimuli and the orienting response of the cat, while others were not (Steinfels et al., 1983). Romo and Schultz (1990) reported several types of DA neurons with different temporal profiles of activation in response to self-initiated arm movements. Similar results were observed by Schultz et al. (1983). It has been reported that the duration of action potential and the sensitivity of D2 receptor-mediated autoinhibition of DA neurons depend on their target structures (Margolis et al., 2008). Behaviorally, more definitive evidence of target structure dependence of DA firing properties was observed by Malenka’s group (Lammel et al., 2012). They found that depending on their target structures, DA neurons were active by rewarding or aversive stimulus. These two groups of neurons were aggregated at different parts of the midbrain and their input sources and output targets were clearly separated. More cellular level diversities of DA neurons have been described (Roeper, 2013). Even with the amount of reported diversity, the impression of a homogeneous population of DA neurons in the midbrain (e.g., Fiorillo et al., 2013) still exists. It is partly due to a historical reason. Up until recently, a common way of identifying midbrain DA neurons in behaving animals was by their long spike shape, their low firing frequency (Grace and Bunney, 1980; Steinfels et al., 1981) and their response to reward. Because one of the criteria of identifying DA neurons is their response to reward (a case of circular logic), most *in vivo* electrophysiological studies (including Hong et al., 2011) would have missed those DA neurons that do not respond to reward, for the lack of means to convince the reviewer/reader of the journal. More careful but cumbersome methods also have been used (e.g., Steinfels et al., 1981; Freeman et al., 1985). It is expected that with more advanced probing tools, such as optogenetics (e.g., Cohen et al., 2012), we may see more diverse types of DA neurons and related interneurons in the coming years.

## Local dopamine activation

Using a new voltammetry technique, a recent study (Howe et al., 2013) shows that even when an animal is motivated, the level of DA in the striatum is non-homogeneous; some parts increase and other parts decrease. This shows that DA activation is more local than has been assumed. I hypothesize that this is because task-relevant pathways in the striatum receive increased DA, while other NPs, which should be suppressed, receive a decreased level of DA. Consistent with this hypothesis, a study showed that when the saccadic eye movement pathway in the caudate was locally depleted of DA, deficits specific to eye movement functions, such as voluntary, memory guided and visually guided eye movements, occurred (Kato et al., 1995; Kori et al., 1995). Autoradiography and PET imaging studies have shown that the receptors and release of DA are not uniformly distributed or released within the traditional structural striatal subdivisions (Staley and Mash, 1996; Drevets et al., 1999; Gurevich and Joyce, 1999; Martinez et al., 2003). A more recent diffusion-weighted MRI and positron emission tomography study by Tziortzi et al. (2013) reports that a Dextroamphetamine-induced DA release across the striatum was regionally differentiated. Moreover, the homogeneity of the DA release was significantly high within the presumed NPs. Also, it has been reported that DA neurons operate at their threshold of maximal energetic boundary (Bolam and Pissadaki, 2012). Therefore, frequent burst activations of most of DA neurons along the whole medio-lateral axis of midbrain may be wasteful and make DA neurons more prone to death (Bolam and Pissadaki, 2012).

## Facilitating or retarding a pathway

Reward prediction error has been an important topic in DA research (e.g., Schultz, 1997; Hong et al., 2011; Lammel et al., 2012). Many DA neurons phasically increase and decrease their firing rates when the subject perceives rewarding and disappointing situations, respectively (Schultz, 1997). I hypothesize that the NPs that have been consistently activated by DA will be facilitated, as in the classic theories of Hebbian-like learning (Hebb, 1949). Instead of this unidirectional account, some brain regions seem to have a bidirectional mechanism with which increments and decrements in DA facilitate and retard their target NPs, respectively, as in the reward prediction error theory and related theories (e.g., Sutton and Barto, 1998; Hong and Hikosaka, 2011a). Where and how these bidirectional modulation signals, commonly referred to as the reward prediction error, are generated is still debated (e.g., Hong et al., 2011; Cohen et al., 2012; Lammel et al., 2012). It is regarded that the timing of an event may be important in neural plasticity (Pawlak and Kerr, 2008); therefore it is likely that the phasic and tonic activation of DA have complimentary roles in learning (Goto et al., 2007). While the details of this topic are beyond the scope of this review, how the background level and phasic supply of DA contribute to learning has been studied (e.g., Frank, 2005; Hong and Hikosaka, 2011a). Though I have emphasized the learning aspects of DA here, there seem to be innate NPs that get mandatory activation by DA (e.g., Steinfels et al., 1983). For example, some DA neurons increase their firing rate when some unexpected stimuli, such as a sudden sound or visual stimulus, are presented (Schultz, 2007). Even for this kind of innate action of DA, there seems to be an adaptive mechanism built into it. For example, some PPTg neurons that are verified to project to DA neurons quickly habituate if the presented stimuli are behaviorally irrelevant (Hong and Hikosaka, 2011b), similarly to the actions of DA neurons (Schultz, 1997).

## Neural pathways in the absence of phasic dopamine input

The number of proposed roles for midbrain DA neurons has been expanding, such as, motor, memory, decision making, reward, aversion and salience (Anstrom and Woodward, 2005; Bromberg-Martin et al., 2010a; Salamone and Correa, 2012). In some cases people have attempted to give a unifying theory under the theme of motivation. However, it has become clear that this characterization of DA fails to cover certain situations. For example, in Parkinson’s disease (PD) patients who have trouble with ordinary daily functions such as walking, the deficits in many cases seem unrelated to motivation itself: often times the patients want (motivation) to walk; it is their motor function that prevents them from walking. In primates (Lynd-Balta and Haber, 1994) and rodents (Ikemoto, 2007), the midbrain DA system is organized in a medio-lateral way: the medial DA area serving the ventromedial striatum and the lateral part serving the dorsolateral part of the striatum (Haber et al., 2000). The striatum itself is organized in a ventromedial-dorsolateral way; ventromedial (limbic), central (associative), and dorsolateral (motor), each area containing parallel NPs (Zahm and Brog, 1992; Pennartz et al., 1994; Parent and Hazrati, 1995; Sidibe et al., 1997; Groenewegen et al., 1999; Haber et al., 2000; Haber, 2003; Haber et al., 2006; Tziortzi et al., 2013). It is known that DA cell death in PD occurs in the lateral part of the SNc (more specifically, ventral tier neurons lying in the most ventral and lateral aspects of SNc (Gibb and Lees, 1991)) first and slowly progresses to the medial part of the SNc (Damier et al., 1999; Ohtsuka et al., 2013). By simply observing the pattern of progression of DA cell degeneration, it is expected that the progression of PD symptoms may start from more motor related dysfunctions to more limbic related problems in a later stage (Braak et al., 2003; Nagai et al., 2012). However, it is also well recognized that PD is a multi-faceted disease with cognitive as well as motor function disturbances even from the beginning (Koller, 1992; Braak et al., 2003; Aarsland et al., 2011). While the degeneration of DA neurons accompanies that of other associated brain regions (Braak et al., 2003), some of the basic neural structures, such as the dorsolateral putamen, in early stages of PD may be preserved even without DA (Kordower et al., 2013). I hypothesize that these DA-depleted brain structures may be in idling states with hyper DA sensitivity and disrupted learning mechanisms (Gerfen, 2003; Hong and Hikosaka, 2011a). Consistent with this hypothesis, L-DOPA, which is known to increase the level of DA in the brain, ameliorates a host of functions in PD patients (Goetz et al., 2005; Pedrosa and Timmermann, 2013). It was reported that there was a virtually complete loss of dopaminergic markers in the postcommissural dorsal putamen in all of their samples about 4 years after the onset of the PD (Kordower et al., 2013). Considering the fact that more than 60% of PD patients after 4–6 years of L-DOPA treatment still see benefits without dyskinesias (Ahlskog and Muenter, 2001), it is clear that tonic level of DA can support a variety of functions in the absence of phasic DA inputs from midbrain DA neurons. This observation is in support of the current theory of DA, which states that DA is there to modulate the level of activity of NPs.

It has been reported that the level of DA in the striatum anti-correlates with beta (and delta) frequency band activities (Costa et al., 2006). Considering the fact that beta (13–30Hz) and lower frequency oscillations (< 13 Hz) in the basal ganglia are antikinetic while higher frequency oscillations (> 60Hz, gamma) are prokinetic (Brown, 2003), the net action of DA seems to be an activating signal for NPs in an idling beta oscillation mode, therefore promoting healthy communications between brain areas. This account of DA activation of NPs supports the current hypothesis of DA.

## The role of the lateral habenula

It is well documented that LHb neurons increase their firing frequency when the subject receives worse than expected results or faces an unpleasant situation (e.g., Matsumoto and Hikosaka, 2007; Hong and Hikosaka, 2008; Matsumoto and Hikosaka, 2009b) and inhibits the midbrain DA neurons (Lisoprawski et al., 1980; Ji and Shepard, 2007; Matsumoto and Hikosaka, 2007) via RMTg (Jhou et al., 2009; Kaufling et al., 2009; Omelchenko et al., 2009; Balcita-Pedicino et al., 2011; Hong et al., 2011). In his detailed review, Hikosaka has proposed that the primary function of the habenula may be to suppress motor activity (Hikosaka, 2010). This conclusion was drawn after observing several facts in different vertebrate species. It is known that virtually all vertebrate species, including those seemingly lacking cognitive functions, have the habenula (Concha and Wilson, 2001). So it is plausible that the LHb plays a direct role in motor suppression in simple vertebrate species. Considering the LHb-DA scheme presented in the current article (Figure 3), it is tempting to propose that the role of the LHb is to suppress the activity of the NPs, including motor NPs, a direct generalization of the hypothesis given by Hikosaka (2010). However, a recent study by Lammel et al. (2012) reports that stimulation of the LHb could activate medial prefrontal cortex (mPFC)-projecting midbrain DA neurons, and condition place avoidance in rats. The activation of DA neurons by the LHb of this experiment defies the generally assumed role of the LHb in suppression of DA neurons, yet reproduces the LHb’s reported role of conditioned avoidance (Stamatakis and Stuber, 2012). The researchers’ DA antagonist experiments targeting the mPFC show that LHb-induced increased DA modulation of the mPFC may be necessary to induce the behavioral conditioning of place avoidance. Considering these results, I propose that the role of the LHb is to suppress maladaptive behaviors and mental processes by suppressing certain NPs and enhancing others via DA modulation.

## Proposed experiments

It has been reported that GPb neurons project to the LHb (Hong and Hikosaka, 2008) and that the LHb inhibits DA neurons in the midbrain via GABAergic neurons in the RMTg (Hong et al., 2011) in monkeys. According to earlier reports by Herkenham and Nauta (1977), it is the lateral part of the LHb that gets the heaviest inputs from the entopeduncular nucleus (EPN) in rodents. In turn, this lateral part of the LHb is the part that projects to the RMTg (see Figure 8, in Herkenham and Nauta (1979) and Kim (2009)). According to my personal experience, this rule seems to apply to monkeys as well; the negative reward prediction error-related LHb neurons are more frequently found in the lateral half of the LHb. Considering the fact that the input and output connectivity of the medial and lateral parts of the LHb are distinct, it must be interesting to see what different functions these two divisions have. By the same token, it is very important to discriminate these two divisions when researchers try to probe the functions of the LHb. For example, a bull’s eye tracer injection into the LHb may include both of these divisions.

Another important piece of information that is currently missing is the scope of LHb modulation of DA neurons. According to current hypotheses, the scale of LHb modulation of DA neurons will determine the scope of LHb modulation of NPs. Hong et al. (2011) reported that over 95% of DA neurons that they examined were modulated by orthodromic stimulations of the RMTg. As mentioned above, however, their criteria of determining DA neurons were biased only to include reward-responding neurons. In addition, it is unknown whether or not all of the RMTg neurons are under the control of the LHb. These crucial questions need to be answered to conclusively clarify the scope of LHb modulation of midbrain DA neurons, and therefore the scope of the NPs that the LHb modulates. Also, it is unknown whether the influence of the LHb on the DA is global or differentiated according to the organization of NPs.

The conventional theory assumes that the pattern of DA neuronal activity is homogeneous across the whole midbrain DA area (e.g., Fiorillo et al., 2013). It is possible that this impression of homogeneity is due to the broad influence of the LHb on the area. One of the crucial features of the current theory is the hypothesis that there are different groups of midbrain DA neurons serving different NPs, potentially driven by the inhibitory feedback from the striosome→DA area along with the excitatory inputs from the PPTg/laterodorsal tagmental area (Kobayashi and Okada, 2007; Sesack and Grace, 2010). Therefore it would be interesting to see whether different NP-specific DA groups get activated depending on their relevance to a given task. This could be achieved by finding different NPs and their counterpart DA neuron groups, and then probing the DA neurons with tasks that could activate only a subset of the NPs at a time.

## Conclusion

The current hypotheses of DA try to answer a more fundamental question of why nature has preserved and advanced this basic DA scheme in many animals. I hypothesize that the DA system is there to recruit and activate only the needed NPs only when they are needed, and also to tune the activity level of mental and behavioral modules, thereby implementing a very efficient resource management system. In terms of learning, the DA evaluation circuit around the basal ganglia may serve to fine-tune the behavior of the animal to increase the chance of survival. This smart management system may have given animals an edge in evolution with adaptive behaviors and a better survival rate in resource (food, water etc.) scarce situations.

## Conflict of interest statement

The authors declare that the research was conducted in the absence of any commercial or financial relationships that could be construed as a potential conflict of interest.
